# Structural basis for the conformational protection of nitrogenase from O_2_

**DOI:** 10.1063/4.0001035

**Published:** 2025-10-27

**Authors:** Sarah M Narehood, Brian D Cook, Suppachai Srisantitham, Vanessa Eng, Angela A Shiau, Kelly L McGuire, R. David Britt, Mark A Herzik, F. Akif Tezcan

**Affiliations:** 1 University of California, San Diego, La Jolla, CA, USA; 2 University of California, Davis, Davis, CA, USA

## Abstract

The low reduction potentials required for the reduction of dinitrogen (N_2_) render metal-based nitrogen-fixation catalysts vulnerable to irreversible damage by dioxygen (O_2_). Such O_2_ sensitivity represents a major conundrum for the enzyme nitrogenase, as a large fraction of nitrogen-fixing organisms are either obligate aerobes or closely associated with O_2_-respiring organisms to support the high energy demand of catalytic N_2_ reduction. To counter O_2_ damage to nitrogenase, diazotrophs use O_2_ scavengers, exploit compartmentalization or maintain high respiration rates to minimize intracellular O_2_ concentrations. A last line of damage control is provided by the ‘conformational protection’ mechanism, in which a [2Fe:2S] ferredoxin-family protein termed

FeSII is activated under O_2_ stress to form an O_2_-resistant complex with the nitrogenase component proteins. Despite previous insights, the molecular basis for the conformational O_2_ protection of nitrogenase and the mechanism of FeSII activation are not understood. Here we report the structural characterization of the *Azotobacter vinelandii* FeSII– nitrogenase complex by cryo-electron microscopy. Our studies reveal a core complex consisting of two molybdenum–iron proteins (MoFePs), two iron proteins (FePs) and one FeSII homodimer, which polymerize into extended filaments. In this three-protein complex, FeSII mediates an extensive network of interactions with MoFeP and FeP to position their iron– sulphur clusters in catalytically inactive but O_2_-protected states. The architecture of the FeSII–nitrogenase complex is confirmed by solution studies, which further indicate that the activation of FeSII involves an oxidation-induced conformational change.

